# Protective Effects of Resveratrol against Chronic Immobilization Stress on Testis

**DOI:** 10.1155/2013/278720

**Published:** 2013-11-06

**Authors:** Gulsah Bitgul, Isil Tekmen, Didem Keles, Gulgun Oktay

**Affiliations:** ^1^Department of Histology and Embryology, Medical Faculty of Dokuz Eylül University, Balcova, 35340 İzmir, Turkey; ^2^Department of Medical Biochemistry, Medical Faculty of Dokuz Eylul University, Balcova, 35340 İzmir, Turkey

## Abstract

*Objective*. The aim of this study was to investigate protective effects of resveratrol, a strong antioxidant, against possible negative effects of chronic immobilization stress on testes of male rats histochemically, immunohistochemically, ultrastructurally, and biochemically. *Material and Methods*. Male Wistar rats were divided into 4 groups (*n* = 7). Group I, control group (C), was not exposed to stress. Group II, stress group (S), was exposed to chronic immobilization stress. In Group III, low dose resveratrol + stress group (LRS), rats were given 10 mg/kg/day resveratrol just before the stress application. In Group IV, high dose resveratrol + stress group (HRS), rats were given 20 mg/kg/day resveratrol just before the stress application. For chronic immobilization stress application animals were put in the plastic tubes (6 cm in diameter, 15 cm in length) during 32 days for 6 hours. All animals were sacrificed 18 hours after the last stress application. *Results*. Histochemical and ultrastructural investigations showed that in stress group there was germ cell deprivation in seminiferous tubules and increase of connective tissue on interstitial area. No significant changes were seen in low and high dose resveratrol groups. After immunohistochemical investigations, TUNEL (+) and Active Caspase-3 (+) cells were increased in seminiferous tubules of stress group compared with those control group, but they were decreased in low and high dose resveratrol groups. According to biochemically results, MDA, GSH, and testosterone levels in stress group showed no significant difference when compared with those of the other groups. *Conclusion*. The chronic immobilization stress increases oxidative stress and apoptosis and causes histological tissue damages; resveratrol can minimize the histological damage in testes significantly.

## 1. Introduction

In this modern world, stress is an unavoidable phenomenon. Stressful situations can lead to many physiological and psychological alterations [1]. Adverse effects of stress on male reproductive system have already been described. Stauber showed how occupational stress in man could affect sperm concentration, motility, and morphology, with these effects being reverted after removal of the stress factor [2]. Apoptotic germ cell death is an important mechanism in testicular development [3] and elimination of germ cells under normal physiological and pathological conditions [4, 5].

It has been show that testicular germ cell apoptosis increases in experimental cryptorchidism [6], local heat stress [7], immobilisation stress [8–13], vasectomy [14], ischemia/reperfusion [15], chronic cigarette smoking [16] and cisplatin, and chronic alcohol [17] treatment. 

Immobilisation stress decreases the activities of catalase, glutathione peroxidase, glutathione transferase, and glutathione reductase in the interstitium of testes. Stress-induced stimulation of the testicular Nitric Oxide (NO) signaling a pathway that leads to the inhibition of both steroidogenic and antioxidant enzymes [18].

It is well known that resveratrol had antioxidant activity in the different stress conditions. However, to our the knowledge, there is no study dealing with the relationship between chronic immobilisation stress-induced apoptosis and resveratrol.

In this study we aimed to investigate protective effects of resveratrol, a strong antioxidant, against possible negative effects of chronic immobilisation stress on testes of male rats histochemically, immunohistochemically, ultrastructurally, and biochemically.

## 2. Materials and Methods

Male Wistar rats, weighing about 200–220 g each, were used in this study. We protected the rights of the animals according to *Guide For the Care and Use of Laboratory Animals* and received approval from *Dokuz Eylul University Local Ethics Committee for Animal Experiments *for our experiment. All animals were subjected to the same conditions; they were maintained at constant temperature (21 ± 2°C) and humidity (50 ± 5%) on a 12-hour light/12-hour dark cycle (light on from 07:00–19:00 h). They were housed in plastic cages (seven rats per cage) and fed on standard pellet food and tap water *ad-libitum*. The animals were divided into four groups (*n* = 7). Group I, control group, was not exposed to stress and allowed to move freely. Group II, stress group, was exposed to chronic immobilization stress. In Group III, low dose resveratrol + stress group, rats were given 10 mg/kg/day resveratrol by intragastric route just before the chronic immobilization stress application. In Group IV, high dose resveratrol + Stress group, rats were given 20 mg/kg/day resveratrol by intragastric route just before the chronic immobilization stress application.

The animals were immobilized inside plastic tubes dimensioned to produce stress without promoting pain (6 cm in diameter × 15 cm long) for 6 h a day over a period of 32 days. Undesirable stress was avoided as much as possible by gentle handling. The rats in groups III and IV were given resveratrol (Cayman Chemicals, USA) 10 mg/kg/day and 20 mg/kg/day, respectively. For group III 100 mg/kg resveratrol and for group IV 200 mg/kg, resveratrol was dissolved in 100% pure ethanol. All experimental animals were sacrificed 18 hours after the last chronic immobilization stress application by giving ether. The testes were removed and dissected free from adjacent connective tissue. Left testis was put in 10% formaldehyde for three days to be fixed, dehydrated in alcohol, and embedded in paraffin. The 5 *μ*m thick sections were cut onto slides. The slides were stained with hematoxylin-eosin, Masson's trichrome, periodic acid schiff (PAS) for histochemical and histometric analysis. The Johnson's testicular biopsy score, basement membrane thickness, and diameter of seminiferous tubule measurements were done for morphometric analysis. For immunohistochemical staining TUNEL and Active Caspase 3 methods were used. A little piece of right testis was fixed with Karnofsky solution for an ultrastructural analysis. The other piece of right testis was used for biochemical tests. Testis was washed with cold normal saline and stored at −80°C. The level of malondialdehyde (MDA) and glutathione (GSH) was measured by using proper kits. The thoracic cavity was opened and blood sample was taken directly from the heart. The sample was taken in a plain vial. Serum was separated by centrifugation and stored at −20°C for testosterone hormone determinations. All the samples were collected in the morning in order to minimize the diurnal variation of testosterone hormone levels. Testosterone estimation was done by ELISA method [19].

The body weights were expressed as the median ± standard deviation (SD). All data were analysed by the nonparametric Kruskal-Wallis test and subsequent individual comparisons by the Mann-Whitney *U*-test. A *P* < 0.05 was considered to be statistically significance.

## 3. Results

In the present study, we observed that body weights of rats in stress group were decreased significantly compared with the control, resveratrol low, and high dose groups (*P* = 0.02). In resveratrol low and high dose groups body weights of rats were increased significantly compared with stress group (*P* = 0.02) (Figure 7(a)). 

**Figure 1:**
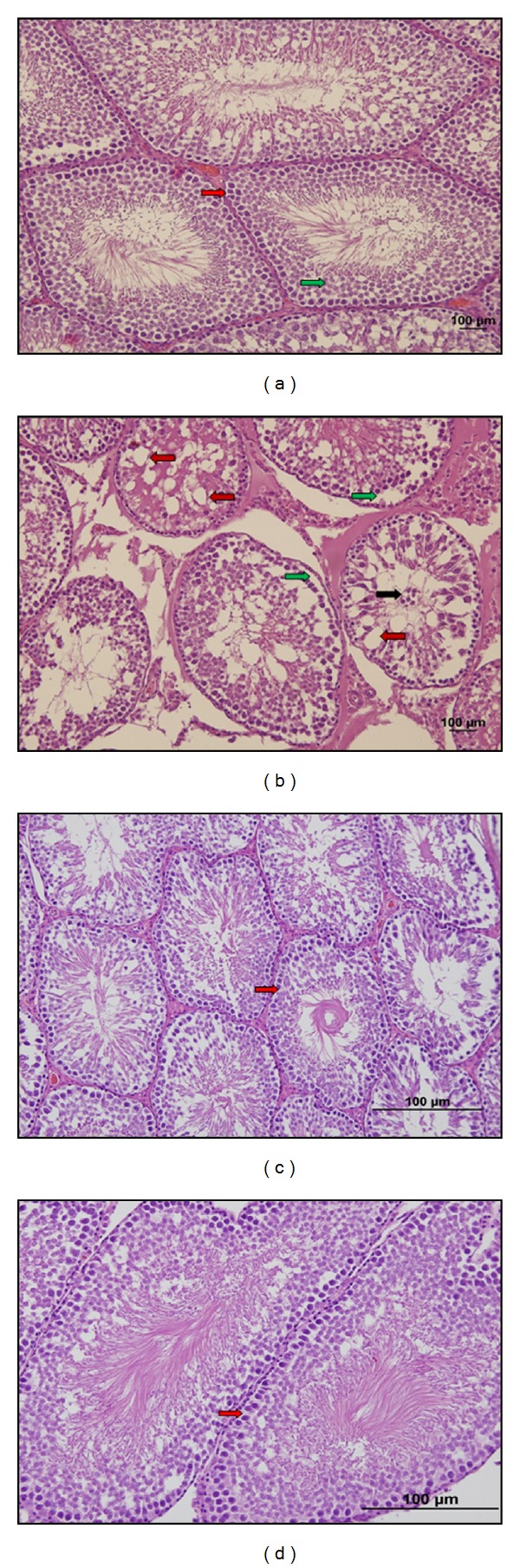
(a) C group. Normal morphology of seminiferous tubules (right red arrow) and inside lining smooth seminiferous epithelium (right green arrow) (hematoxylin-eosin ×20). (b) S group. In the seminiferous tubules there are separations between germ cells and the basement membrane (right green arrow) and gaps (right red arrow), and because of pouring degenerated germ cells debris is observed in the lumen of tubules (right black arrow) (hematoxylin-eosin ×20). (c) LRS group. Damages seen in stress group are greatly reduced (right red arrow) and in the lumen of seminiferous tubules mature spermiums are observed. (Hematoxylin-eosin ×20). (d) HRS group. Morphology of the seminiferous tubules is similar to the control group (right red arrow). (Hematoxylin-Eosin ×20).

**Figure 2:**
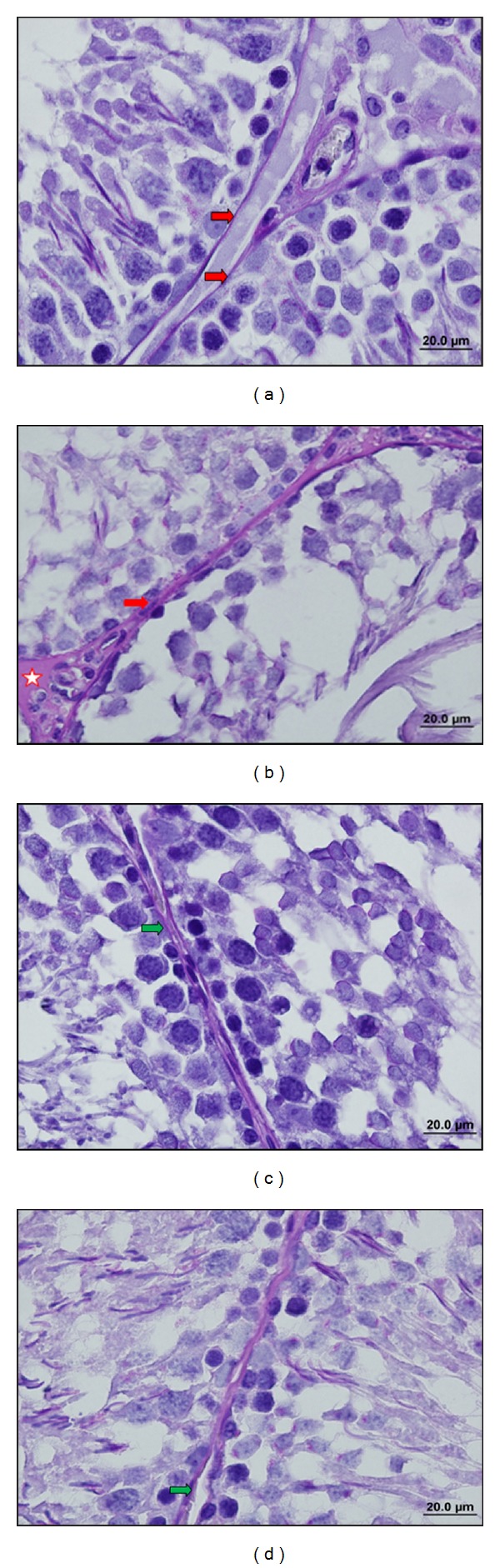
(a) C group. The basal membrane of the seminiferous tubule (right red arrow) has normal thickness (PAS ×100). (b) S group. In interstitial area connective tissue is increased (red bordeed star); diameter of the seminiferous tubule is reduced and basement membrane of seminiferous tubule (right red arrow) has become thicker than that of S group (PAS ×100). (c) LRS group. Basement membrane thickness (right green arrow) and diameter of seminiferous tubules are similar in the C group (PAS ×100). (d) HRS group. Basement membrane thickness (right green arrow) and diameter of seminiferous tubules are similar in the C group (PAS ×100).

**Figure 3:**
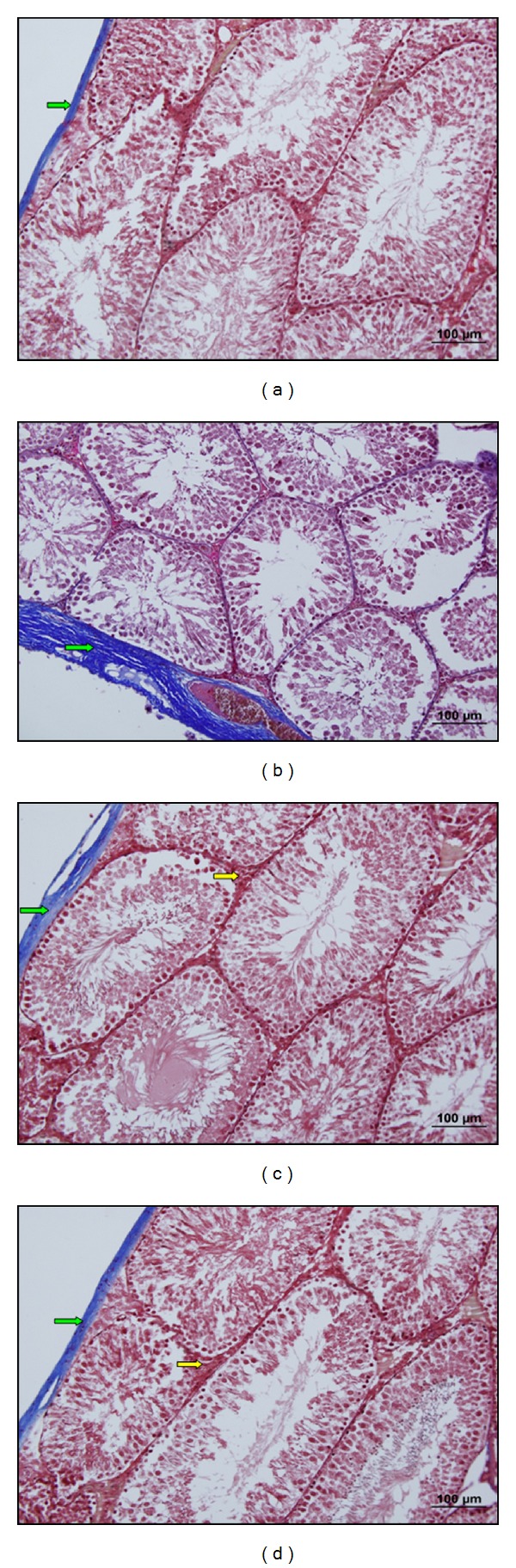
(a) C group. The normal structure of the tunica albuginea (right green arrow) is observed (Masson's Trichrome ×20). (b) S group. The increase in connective tissue of the tunica albuginea (right green arrow) is observed (Masson's Trichrome ×20). (c) LRS group. The normal structure of connective tissue is observed in interstitial area (right yellow arrow) and tunica albuginea (right green arrow) (Masson's Trichrome ×20). (d) HRS group. The normal structure of connective tissue is observed in interstitial area (right yellow arrow) and tunica albuginea (right green arrow) (Masson's Trichrome ×20).

In control group, the seminiferous tubules were bounded together by loose intertubular connective tissue, which contained fibroblasts, collagen fibers, blood vessels, and groups of interstitial cells or leydig cells. These cells were large and polyhedral with euchromatic nucleus, containing nucleoli. The cytoplasm was scanty and poorly stained. The capillaries were infiltrated among the clumps of leydig cells (Figures 1(a), 2(a), 3(a), and 6(a)).

In stress group, there was a marked reduction in spermatogenesis. The architecture of the testis was maintained, but the germinal epithelium showed disorganization as well as marked degenerative changes. There was cell debris in the lumen of tubules as a result of infusion of degenerated germ cells. The basement membrane of the tubules was thicker than the control group. As the seminiferous tubules were reduced in diameter, the interstitial spaces were increased. The numbers of fibroblasts as well as of collagen fibers were also increased. The cell membrane of leydig cells was poorly defined, with cytoplasmic vacuolations in many cells. The nuclei were circular to oval in shape and were smaller as compared to control groups. The capillary network was well defined. Fairly a good number of fat cells with large clear spaces and small eccentric nucleus were observed among the clumps of leydig cells. Ultrastructural observations showed that in stress group there was a distinct degeneration in seminiferous epithelium. Basement membrane was thicker than the control group. Also between Sertoli and germ cells there was a distinct separation (Figures 1(b), 2(b), 3(b), 6(b), 7(c), and 7(d)).

In resveratrol low and high dose groups, we observed that the shape and diameter of seminiferous tubules were the same as those of control group. The thickness of basement membrane was reduced compared with that of stress group. The germ cells in the tubules were arranged in an order like control group. The degenerations seen in stress group were disappeared (Figures 1(c), 3(c), 6(c), 1(d), 3(d), and 6(d)).

As a result of the Johnson's testicular biopsy score, there was a significant decrease in stress group compared with the control group (*P* = 0.002). In resveratrol low and high dose groups, testicular biopsy score means were increased significantly compared with those of the stress group (*P* = 0.002) (Figure 7(b)).

**Figure 4:**
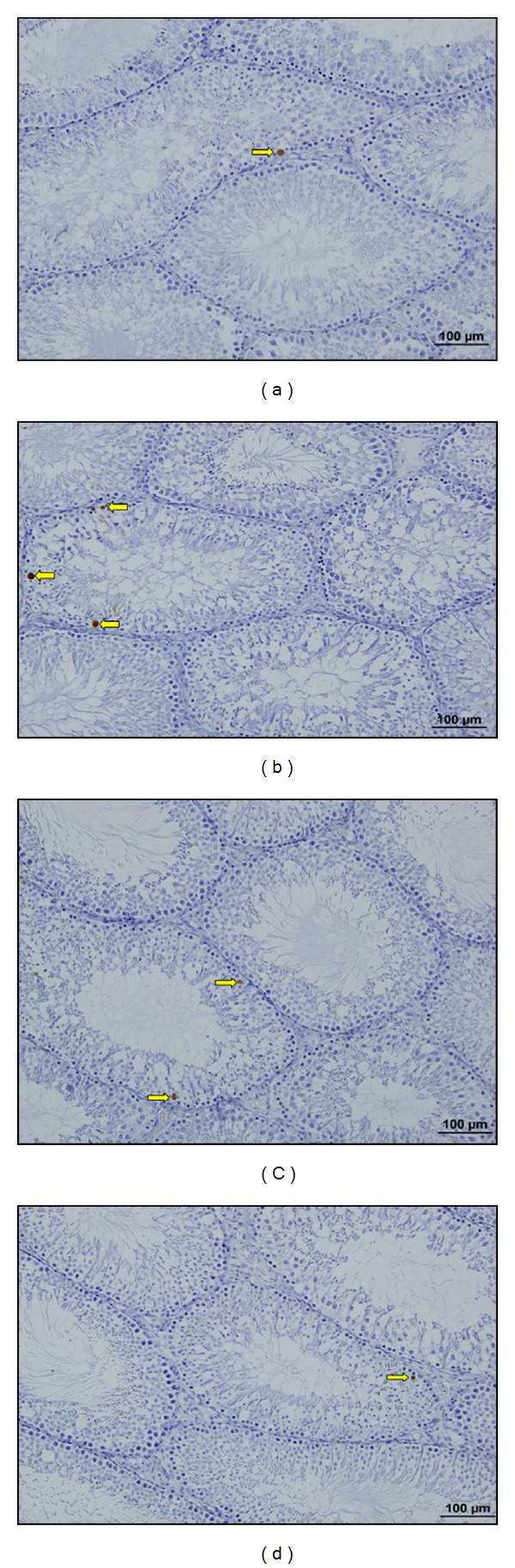
(a) C group. TUNEL (+) cells (right yellow arrow) are observed (TUNEL ×20). (b) S group TUNEL (+) cells (right yellow arrow) are observed. (TUNEL ×20). (c) LRS group. TUNEL (+) cells (right yellow arrow) are observed (TUNEL ×20). (d) HRS group. TUNEL (+) cells (right yellow arrow) are observed (TUNEL ×20).

**Figure 5:**
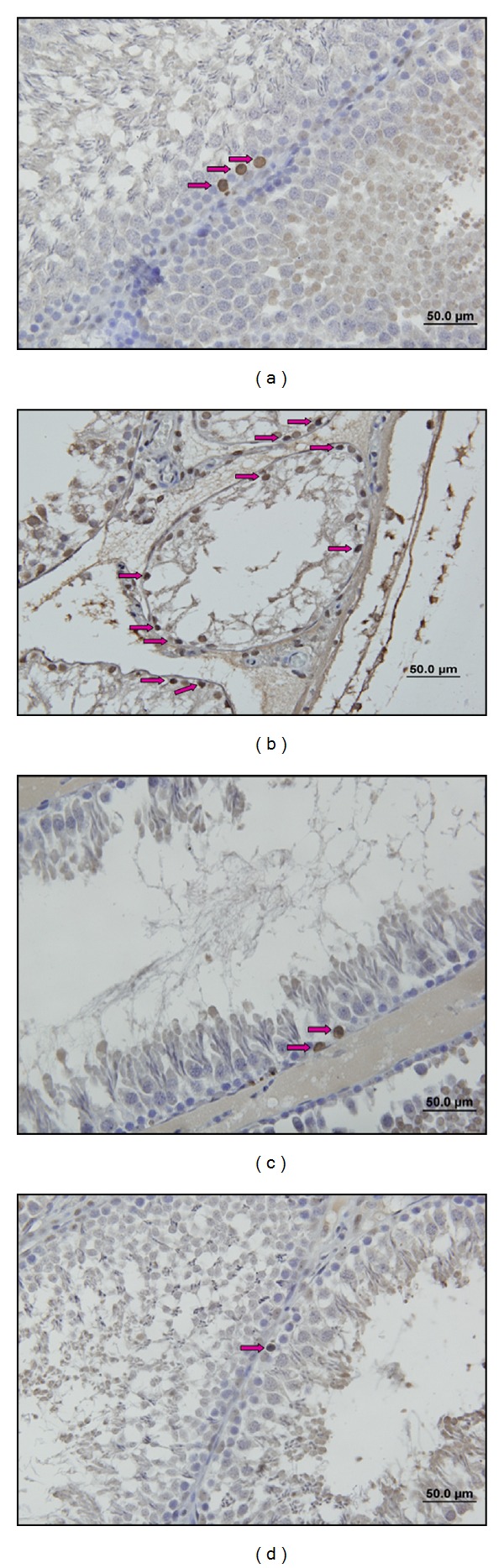
(a) C group. Active caspase-3 (+) cells are (right pink arrow) observed (Active Caspase-3 ×40). (b) S group. Active caspase-3 (+) cells are (right pink arrow) observed (Active Caspase-3 ×40). (c) LRS group. Active caspase-3 (+) cells are (right pink arrow) observed (Active Caspase-3 ×40). (d) HRS group. Active caspase-3 (+) cells are (right pink arrow) observed (Active Caspase-3 ×40).

**Figure 6:**
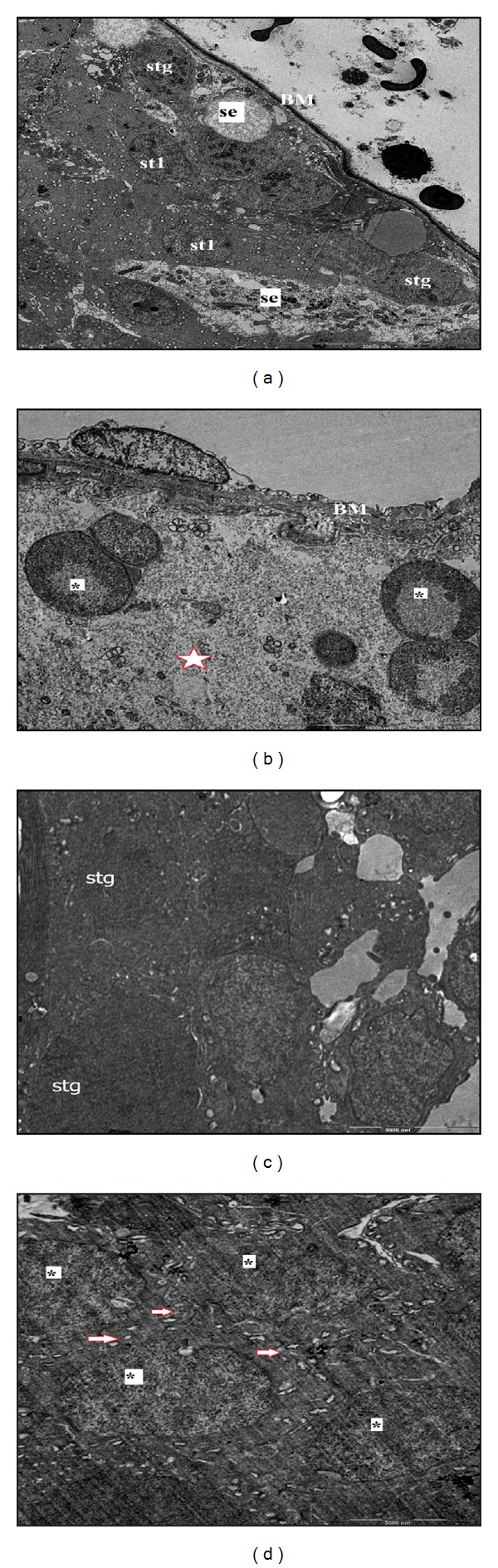
(a) C group. Spermatogonium (stg) and sertoli cells (se) sitting on properly structured basement membrane (BM) and above primer spermatoyte (st1) are observed (Uranyl acetate-lead citrate ×1250). (b) S group. The basement membrane (BM) is thicker and the gaps (red bordeed star) between germ cells are observed (Uranyl acetate-lead citrate ×1250) (c) LRS group. The seminiferous tubule epithelium is similar to the control group (Uranyl acetate-lead citrate ×1250). (d) HRS group. The seminiferous tubule epithelium is similar to the control group and cell-cell connection (right red borded arrow) is observed. (Uranyl acetate-lead citrate ×1250).

**Figure 7:**
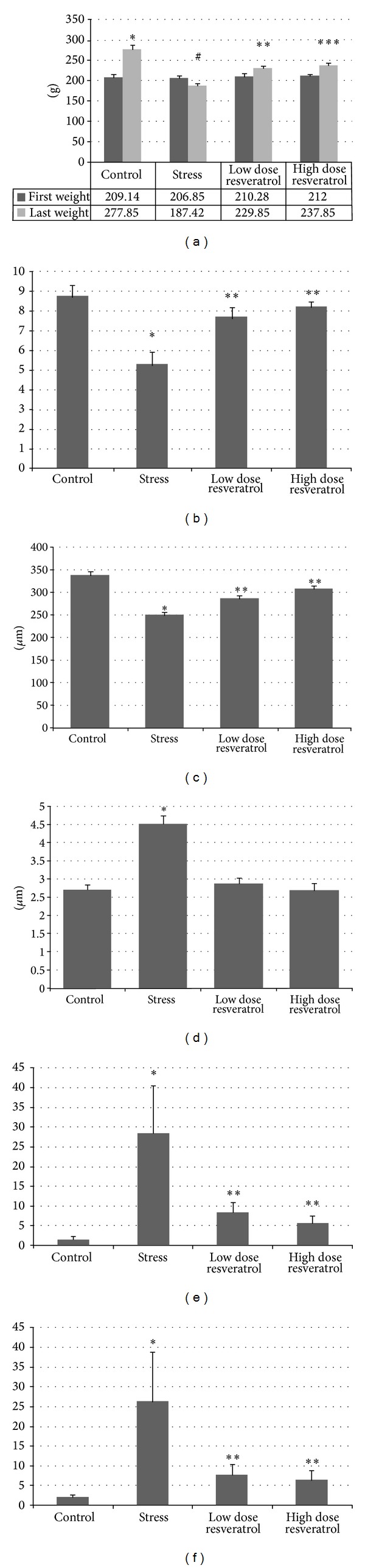
(a) Changes in rat weights. #: significant differences versus control. ∗, ∗∗, ∗∗∗: significant differences versus first weights. (b) Johnson's testicular biopsy score. ∗: significant decrease versus control. ∗∗: significant increase versus stress. (c) Changes in diameter of seminiferous tubules. ∗: significant decrease versus control. ∗∗: significant increase versus stress. (d) Changes in thickness of basement membrane. ∗: significant increase versus control. (e) Number of TUNEL (+) cells. ∗: significant increase versus control. ∗∗: significant decrease versus stress. (f) Number of Active Caspase-3 (+) cells. ∗: significant increase versus control. ∗∗: significant decrease versus stress.

After immunohistochemical investigations, we observed that apoptotic cell death was increased significantly in stress group compared with control group (*P* = 0.001) (Figures 4(a)–4(d) and 5(a)–5(d)). In low and high dose resveratrol groups, TUNEL (+) cells and Active Caspase 3 (+) cells were decreased significantly compared with stress group, (*P* = 0.001). However, there was no significant difference between low and high dose resveratrol groups (*P* = 0.3) (Figures 7(e) and 7(f)).

There was no a significant difference between the four groups when compared in the way of MDA and GSH levels in testicular tissue (*P* = 0.89). Besides, serum testosterone levels showed no significant difference in stress group when compared with that of control and resveratrol low dose groups (*P* = 0.48). In resveratrol high dose group, serum testosterone level showed a significant increase when compared with that of stress group (*P* = 0.02).

## 4. Discussion

Stress can be described as the sum total of all the reactions of the body, which disturb the normal physiological equilibrium and result in a state of threatened homeostasis. Stress is an internationally recognized phenomenon fortified by advancement of industrialization and a demanding civilization. Thus, every person today faces stressful situations in day-to-day life [20].

Stress represents reaction of body to stimuli that tend to disturb its normal physiological equilibrium or homeostasis and has been defined as nonspecific response of the body to any demand imposed on it. Stress, depression, and associated mental health problems have increased tremendously in modern times. Several studies have suggested that stress may cause infertility by affecting the gonads [21].

The effects of stress that have formed on people are examined on animals by applying a variety of methods. Immobilization stress method causes both psychological and physiological stress by the restriction of movement, aggression, feeling of distress, and burnout. Consequently, the immobilization stress model is considered as an easy and convenient method [13].

As a result of a variety of studies, it has been indicated that stress induces free radical formation and oxidative tissue damages [22]. To reduce the effects of stress, which cause many diseases, substances showing antioxidant properties were carried out on [21].

In this study, we aimed to investigate protective effects of resveratrol, a strong antioxidant, against possible negative effects of chronic immobilization stress on testes of male rats histochemically, immunohistochemically, ultrastructurally, and biochemically.

Herman et al. have found that weight loss has occurred as a result of stress. The reason of weight loss is explained as a result of reduced feed and water consumption or exhaustion of body reserves as a result of increased metabolic activity [23]. In our study, we observed weight loss in stress group in line with the literature. While there was a statistically significant increase between initial and final weights of the subjects in the C, LRS, and HRS groups, there was a statistically significant decrease between initial and final weights in the S group. We believe that the reason of weight loss is reduced feed and water consumption or exhaustion of body reserves as a result of increased metabolic activity. We think that resveratrol, used as an antioxidant, reduced the excessive metabolic activity caused by stress or increased water and feed consumption in LRS and HRS groups compared to S group.

In recent years, the effects of the psychological and physiological stress on male infertility have been researched. As a result it is reported that, stress causes endocrine disorders in testes and damages in testicular morphology. Pook et al. showed in their study that psychological stress in humans reduces the movement of the sperm and sperm quality which leads to infertility [24]. Rai et al. clearly demonstrated that immobilization stress disturbs spermatogenic and endocrine function of testes [19]. Yazawa et al. reported that stress influences male reproductive activity and increases the apoptotic index in the seminiferous tubules of the rat testes. Apoptotic germ cells, most frequently spermatogonia and primary spermatocytes, were detected in the testes of immobilised rats for 2 h daily on 7 consecutive days [13]. In parallel with these studies, we observed widely tubule damages histochemically and developed apoptosis immunohistochemically in testes of rats exposed to chronic immobilization stress.

It is considered that the increase in the level of blood serum cortisol is an indicator of organism's response to stress [13, 25]. Andersen et al. have used many different animal models of stress (physical inactivity, buoyancy, insomnia, and cold application), and they have reported that immobilization stress model increases cortisol levels and decreases testosterone levels as the other models. They have showed that this hormonal profile disrupts testicular function [26]. Palma et al. applied 1-hour acute immobilization stress in their study. They have reported a significant increase in blood cortisol levels which is an indicator of stress [27]. In stress situations, secretion of glucocorticoids increases and inhibits the reproduction. Thus it is thought that stress inhibits reproduction by way of the glucocorticoids. Tilbrook et al. have showed that in prolonged stress gonadotropin secretion is suppressed; therefore reproduction is inhibited [28].

There are some other studies that show increased glucocorticoid levels in the bloodstream during stress [13, 25]. Yazawa et al. applied immobilization stress for 2 h daily on 7 consecutive days to rats. They have observed that there was an increase in serum cortisol levels and a decrease in testosterone levels of stress group [13]. Demura et al. studied the endocrinological response of acute immobilization stress and reported a decrease in plasma testosterone level [10]. Akinbami et al. also reported a decrease in plasma testosterone level after acute immobilization stress [29]. Kostić et al. reported that acute immobilization stress causes significant decrease in serum testosterone level [30]. As a result of the literature research, we found that acute stress causes a decrease in testosterone levels. Pollard et al. reported significant elevation in serum testosterone level following a prolonged exposure to psychological stress. He believed that the decreased hepatic clearance of testosterone could be responsible for it [31]. In our study, we observed no significant difference between C and S groups. Low testosterone levels, observed in acute stress, could not be observed after chronic stress. In this case we suggest that the subjects showed a physiological adaptation to chronic stress. In LRS group, there was a statistically significant decrease in serum testosterone levels compared with those of the S group. However in HRS group there was a statistically significant increase in serum testosterone levels compared with those of the S group. As a result; we are of the opinion that high dose resveratrol is more defensive than low dose resveratrol against the chronic stress. 

Gao et al. showed glucocorticoids control mitosis and apoptosis induction in testicular cells [25]. Yazawa et al. used TUNEL method to show advanced apoptosis in series of spermatogenic cells after chronic stress [13]. In other study, Yazawa et al. gave glucocorticoids from the outside to rats and showed the increase in the level of the cortisol in blood. As a result of this, they showed germ cell apoptosis using TUNEL method again [32]. In our study, in order to show apoptosis in testis immunohistochemically we used TUNEL and Active Caspase-3 staining methods. As a result of exposure to chronic stress, apoptosis was significantly higher in stress group than in control group. In stress group, the number of TUNEL (+) and Caspase 3 (+) cells was increased significantly than the cells in control group. The results of our study support the literature.

In the literature, it has been shown that there is a relationship between immobilization stress, lipid peroxidation, and protein oxidation. Baraboǐ reported in his study that immobilization stress increases the synthesis of corticosterone, serotonin, and catecholamine hormones which play a role in stress, and these hormones make tissue injury by inducing lipid peroxidation in various organs [22]. Liu et al. examined the effect of immobilized stress-induced oxidative stress in lipid, protein, and DNA damage of the rat brain [33]. Davydov and Shvets examined the activity of lipid peroxidation in the hearts of adult and old rats during immobilization stress [34]. Sosnovskiĭ et al. studied the effects of stress on the brain, liver, stomach, and red blood cells using a variety of stress models, and the mechanisms of tissue damage [35]. Şahin and Gümüşlü examined the effects of immobilization stress on antioxidants, protein, and lipid peroxidation of various tissues [36]. In our study, we showed the testicular damage, caused by chronic immobilization stress, by measuring the levels of MDA (Malondialdehyde) as a marker of oxidative stress. Our findings are not statistically significant. In the literature, there is not any data evaluating the level of MDA after testicular damage caused by chronic immobilization stress. 

GSH (Glutathione) is a main product that occurs after oxidative cell injury. The decrease in GSH levels in tissues is an important factor that shows the tissue damage and lipid peroxidation [37]. Musch et al. stated that the agents affect the lifestyle adversely and that oxidants under the influence of stress reduces the level of cellular GSH [38]. Seçkin et al. showed the decrease in the activity of antioxidant enzymes during stress period in their study [39]. Şahin and Gümüşlü reported the increased MDA levels and decreased GSH levels in brain and stomach of rats in stress group [36]. In our study, we measured the levels of GSH produced in the testis against the damage of chronic immobilization stress. Our findings are not statistically significant. In the literature, there is not any data evaluating the level of GSH after testicular damage caused by chronic immobilization stress. 

Resveratrol has strong effects as an antioxidant and is commonly used in recent studies. Juan et al. showed that transresveratrol has a therapeutic effect on spermatogenesis and testis of the adult rats. They also reported that there is an increase in the production of spermatozoa in rats with oral resveratrol treatment [40]. In parallel with the literature, we observed an increase in the production of spermatozoa in resveratrol groups compared to that of the stress group. In various experimental models, antiapoptotic effect of resveratrol is shown [41, 42]. In our study, we observed that in resveratrol groups the number of TUNEL (+) and Active Caspase-3 (+) cells was decreased significantly compared to that of the stress group.

Wang et al. showed in their study that resveratrol increases levels of GSH in tissues [43]. In testicular ischemia model Uguralp et al. indicated that resveratrol increases GSH levels [44]. Sener et al. reported that resveratrol increases tissue GSH levels after ischemia [45]. Botelho et al. observed that resveratrol had no effect on markers of oxidative stress [46]. In parallel with the literature, as a result of our study, we did not observe any positive effect of resveratrol on MDA and GSH levels in testis.

Ultrastructurally, we showed that resveratrol prevented the testicular damage caused by stress. Testicular morphology observed in resveratrol groups is similar to that observed in the control group.

## 5. Conclusion

This study has demonstrated that resveratrol prevented the negative effects of chronic immobilization stress on testes of male rats.

## References

[1] Koolhaas JM, Meerlo P, De Boer SF, Strubbe JH, Bohus B (1997). The temporal dynamics of the stress response. *Neuroscience and Biobehavioral Reviews*.

[2] Stauber M (1973). Männliche Fertilitätsstörungen Durch Beruflichen stress. *Medizinische Monatsschrift*.

[3] Ketola I, Toppari J, Vaskivuo T, Herva R, Tapanainen JS, Heikinheimo M (2003). Transcription factor GATA-6, cell proliferation, apoptosis, and apoptosis-related proteins Bcl-2 and Bax in human fetal testis. *Journal of Clinical Endocrinology and Metabolism*.

[4] Sinha H, Swerdloff R (1999). Hormonal and genetic control of germ cell apoptosis in the testis. *Reviews of Reproduction*.

[5] Richburg JH (2000). The relevance of spontaneous- and chemically-induced alterations in testicular germ cell apoptosis to toxicology. *Toxicology Letters*.

[6] Yin Y, Hawkins KL, Dewolf WC, Morgentaler A (1997). Heat stress causes testicular germ cell apoptosis in adult mice. *Journal of Andrology*.

[7] Yamamoto CM, Sinha Hikim AP, Huynh PN (2000). Redistribution of Bax is an early step in an apoptotic pathway leading to germ cell death in rats, triggered by mild testicular hyperthermia. *Biology of Reproduction*.

[8] Akinbami MA, Philip GH, Sridaran R, Mahesh VB, Mann DR (1999). Expression of mRNA and proteins for testicular steroidogenic enzymes and brain and pituitary mRNA for glutamate receptors in rats exposed to immobilization stress. *Journal of Steroid Biochemistry and Molecular Biology*.

[9] Almeida SA, Petenusci SO, Franci JAA, Rosa E Silva AAM, Lamano Carvalho TL (2000). Chronic immobilization-induced stress increases plasma testosterone and delays testicular maturation in pubertal rats. *Andrologia*.

[10] Demura R, Suzuki T, Nakamura S, Komatsu H, Odagiri E, Demura H (1989). Effect of immobilization stress on testosterone and inhibin in male rats. *Journal of Andrology*.

[11] Pellegrini A, Grieco M, Materazzi G, Gesi M, Ricciardi MP (1998). Stress-induced morphohistochemical and functional changes in rat adrenal cortex, testis and major salivary glands. *Histochemical Journal*.

[12] Suárez M, Fiol de Cuneo M, Vincenti L, Ruiz RD (1996). Changes in corticosterone levels and sperm functional activity by chronic stress in rats. *Archives of Physiology and Biochemistry*.

[13] Yazawa H, Sasagawa I, Ishigooka M, Nakada T (1999). Effect of immobilization stress on testicular germ cell apoptosis in rats. *Human Reproduction*.

[14] Shiraishi K, Naito K, Yoshida K-I (2001). Vasectomy impairs spermatogenesis through germ cell apoptosis mediated by the p53-Bax pathway in rats. *Journal of Urology*.

[15] Lysiak JJ, Turner SD, Turner TT (2000). Molecular pathway of germ cell apoptosis following ischemia/reperfusion of the rat testis. *Biology of Reproduction*.

[16] Rajpurkar A, Jiang Y, Dhabuwala CB, Dunbar JC, Li H (2002). Cigarette smoking induces apoptosis in rat testis. *Journal of Environmental Pathology, Toxicology and Oncology*.

[17] Zhu Q, Meisinger J, Emanuele NV, Emanuele MA, LaPaglia N, Van Thiel DH (2000). Ethanol exposure enhances apoptosis within the testes. *Alcoholism: Clinical and Experimental Research*.

[18] Kostic TS, Andric SA, Maric D, Kovacevic RZ (2000). Inhibitory effects of stress-activated nitric oxide on antioxidant enzymes and testicular steroidogenesis. *Journal of Steroid Biochemistry and Molecular Biology*.

[19] Rai J, Pandey S, Srivastava R (2004). Testosterone hormone level in albino rats following restraint stress of long duration. *Journal of the Anatomical Society of India*.

[20] Chrousos GP, Gold PW (1992). The concepts of stress and stress system disorders: overview of physical and behavioral homeostasis. *Journal of the American Medical Association*.

[21] Rai D, Bhatia G, Palit G, Pal R, Singh S, Singh HK (2003). Adaptogenic effect of Bacopa monniera (Brahmi). *Pharmacology Biochemistry and Behavior*.

[22] Baraboǐ VA (1989). The role of lipid peroxidation in the mechanism of stress. *Fiziologicheskii Zhurnal*.

[23] Herman JP, Adams D, Prewitt C (1995). Regulatory changes in neuroendocrine stress-integrative circuitry produced by a variable stress paradigm. *Neuroendocrinology*.

[24] Pook M, Tuschen-Caffier B, Krause W (2004). Is infertility a risk factor for impaired male fertility?. *Human Reproduction*.

[25] Gao HB, Tong MH, Hu YQ, Guo QS, Ge R, Hardy MP (2002). Glucocorticoid induces apoptosis in rat Leydig cells. *Endocrinology*.

[26] Andersen ML, Bignotto M, Machado RB, Tufik S (2004). Different stress modalities result in distinct steroid hormone responses by male rats. *Brazilian Journal of Medical and Biological Research*.

[27] Palma BD, Suchecki D, Tufik S (2000). Differential effects of acute cold and footshock on the sleep of rats. *Brain Research*.

[28] Tilbrook AJ, Turner AI, Clarke IJ (2000). Effects of stress on reproduction in non-rodent mammals: the role of glucocorticoids and sex differences. *Reviews of Reproduction*.

[29] Akinbami MA, Taylor MF, Collins DC, Mann DR (1994). Effect of a peripheral and a central acting opioid antagonist on the testicular response to stress in rats. *Neuroendocrinology*.

[30] Kostić T, Andrić S, Kovačević R, Marić D (1997). The effect of opioid antagonists in local regulation of testicular response to acute stress in adult rats. *Steroids*.

[31] Pollard I, Bassett JR, Joss JMP (1980). Plasma testosterone levels and Δ^5^-3*β*-hydroxysteroid dehydrogenase activity in the testis of the rat following prolonged exposure to stress. *Journal of Reproduction and Fertility*.

[32] Yazawa H, Sasagawa I, Nakada T (2000). Apoptosis of testicular germ cells induced by exogenous glucocorticoid in rats. *Human Reproduction*.

[33] Liu J, Wang X, Shigenaga MK, Yeo HC, Mori A, Ames BN (1996). Immobilization stress causes oxidative damage to lipid, protein, and DNA in the brain of rats. *FASEB Journal*.

[34] Davydov VV, Shvets VN (2001). Lipid peroxidation in the heart of adult and old rats during immobilization stress. *Experimental Gerontology*.

[35] Sosnovskiĭ A, Blashova T, Prigova G, Pertsov S, Kubatiev AA, Pertsov SS (1993). Antioxidant enzymatic activity in the limbic-reticular structures of the rat brain after short-term immobilization. *Bjulleten' Eksperimental'noj Biologii i Mediciny*.

[36] Şahin E, Gümüşlü S (2007). Immobilization stress in rat tissues: alterations in protein oxidation, lipid peroxidation and antioxidant defense system. *Comparative Biochemistry and Physiology Part C*.

[37] Lomaestro BM, Malone M (1995). Glutathione in health and disease: pharmacotherapeutic issues. *Annals of Pharmacotherapy*.

[38] Musch MW, Walsh-Reitz MM, Chang EB (2006). Roles of ZO-1, occludin, and actin in oxidant-induced barrier disruption. *American Journal of Physiology*.

[39] Seçkin Ş, Alptekin N, Doğru-Abbasoğlu S, Koçak-Toker N, Toker G, Uysal M (1997). The effect of chronic stress on hepatic and gastric lipid peroxidation in long-term depletion of glutathione in rat. *Pharmacological Research*.

[40] Juan ME, González-Pons E, Munuera T, Ballester J, Rodríguez-Gil JE, Planas JM (2005). trans-Resveratrol, a natural antioxidant from grapes, increases sperm output in healthy rats. *Journal of Nutrition*.

[41] Jiang Y-G, Peng T, Luo Y, Li M-C, Lin Y-H (2008). Resveratrol reestablishes spermatogenesis after testicular injury in rats caused by 2,5-hexanedione. *Chinese Medical Journal*.

[42] Uguralp S, Usta U, Mizrak B (2005). Resveratrol may reduce apoptosis of rat testicular germ cells after experimental testicular torsion. *European Journal of Pediatric Surgery*.

[43] Wang J, He D, Zhang Q, Han Y, Jin S, Qi F (2009). Resveratrol protects against cisplatin-induced cardiotoxicity by alleviating oxidative damage. *Cancer Biotherapy and Radiopharmaceuticals*.

[44] Uguralp S, Mizrak B, Karabulut AB (2005). Resveratrol reduces ischemia reperfusion injury after experimental testicular torsion. *European Journal of Pediatric Surgery*.

[45] Sener G, Tuğtepe H, Yüksel M, Cetinel S, Gedik N, Yeğen BC (2006). Resveratrol improves ischemia/reperfusion-induced oxidative renal injury in rats. *Archives of Medical Research*.

[46] Botelho GGK, Bufalo AC, Boareto AC (2009). Vitamin C and resveratrol supplementation to rat dams treated with di(2-ethylhexyl)phthalate: impact on reproductive and oxidative stress end points in male offspring. *Archives of Environmental Contamination and Toxicology*.

